# Primary Tumor Resection Provides Survival Benefits for Patients with Synchronous Brain Metastases from Colorectal Cancer

**DOI:** 10.3390/diagnostics12071586

**Published:** 2022-06-29

**Authors:** Xiaofei Cheng, Yanqing Li, Dong Chen, Xiangming Xu, Fanlong Liu, Feng Zhao

**Affiliations:** 1 Department of Colorectal Surgery, The First Affiliated Hospital, Zhejiang University School of Medicine, Hangzhou 310003, China; xfcheng@zju.edu.cn (X.C.); cdking@zju.edu.cn (D.C.); zjxxm1974@163.com (X.X.); 2 Department of Pathology, The Second Affiliated Hospital Zhejiang University School of Medicine, Hangzhou 310009, China; 2512074@zju.edu.cn; 3 Department of Radiation Oncology, The First Affiliated Hospital, Zhejiang University School of Medicine, Hangzhou 310003, China

**Keywords:** colorectal cancer (CRC), brain metastases (BMs), survival, prognosis factors, primary tumor resection (PTR)

## Abstract

Background: Brain metastases (BMs), particularly synchronous brain metastases, in colorectal cancer (CRC) patients are uncommon. The survival benefit of primary tumor resection (PTR) in patients with metastatic colorectal cancer is controversial. Whether PTR can bring survival benefits to patients with BMs of CRC has not been reported. Methods: From 2010 to 2016, 581 CRC patients with BMs from the Surveillance, Epidemiology, and End Results (SEER) database were divided into PTR and non-PTR groups. The log-rank test was used to compare the survival distributions. The Kaplan-Meier method was used to estimate survival. By controlling additional prognostic factors, a Cox proportional multivariate regression analysis was used to estimate the survival benefit of PTR. Results: The median overall survival for CRC patients with synchronous BMs was 3 months, with a 1-year survival rate of 27.2% and a 2-year survival rate of 12.8%. The PTR group contained 171 patients (29.4%), whereas the non-PTR group had 410 patients (70.6%). Patients who underwent PTR had a 1-year survival rate of 40.2% compared to 21.7% in those who did not (*p* < 0.0001). Cox proportional analysis showed that patients ≥60 years (hazard ratio [HR] 1.718, 95% confidence interval [CI] 1.423–2.075, *p* < 0.0001) had a shorter OS than patients < 60 years of age. OS was better in CEA-negative than in CEA-positive patients (HR 0.652, 95% CI 0.472–0.899, *p* = 0.009). Patients in whom the primary tumor was removed had considerably improved prognoses (HR 0.654, 95% CI 0.531–0.805, *p* < 0.0001). Subgroup analysis revealed that the PTR group achieved a survival advantage except for patients with CEA negative. Conclusions: Patients with synchronous BMs from CRC may benefit from primary tumor resection (PTR). Age, CEA level, and PTR were independent prognostic risk factors for CRC patients with synchronous BMs.

## 1. Introduction

Colorectal cancer (CRC) is the main cause of cancer-related death globally, with mortality rates of 45%, 35%, and 47.8% in Europe, the United States, and worldwide, respectively [1]. The main cause of disease progression and death for CRC patients is metastasis, which is present in ~25% of patients at the time of diagnosis and develops in another 25% later in life [2]. The most frequent sites of metastasis in CRC are the liver and lungs. Brain metastases (BMs) are rare, with an incidence of only 0.3–9% [3]. However, the prevalence of BMs may be rising over time, owing to improved CRC diagnosis at an earlier stage, thorough follow-up, and higher patient survival as a result [4]. Furthermore, brain or cerebral imaging is not usually suggested in clinical recommendations concerning metastatic CRC at risk, which means that BMs are commonly detected only when symptoms arise, which is typically late in the disease process. Headaches, nausea, and hemiparesis are common symptoms of BMs, and they contribute to a poor quality of life [5]. As a result, CRC BMs cannot be dismissed as a minor clinical issue that requires little attention.

The prognosis of patients with BMs from CRC is very poor, as the median survival is only 2–9.6 months [6,7,8]. Age, Karnofsky performance status (KPS), extracerebral metastases, and BMs number are predictive variables for OS in retrospective investigations [9,10,11,12]. CRC BMs patients with negative CEA levels had a 3-month survival advantage, implying that a positive CEA level is related to a poor OS [6,13]. The prognosis of CRC BMs is also affected by the location of the underlying tumor. Compared to patients with left-sided CRC, those with right-sided CRC develop BMs much earlier and have a significantly worse prognosis [14]. The treatment of metastatic CRC has altered substantially in recent years, owing mostly to the development of targeted treatments and immunotherapy. While there is no consensus on how to treat patients with CRC BMs, their care is similar to that of patients with other solid tumors. It is based on criteria such as the patient’s performance status, the underlying tumor’s features, and the number/location of the brain lesions [15]. The goal of treatment is typically palliative, and alternatives include surgical excision, stereotactic radiosurgery (SRS), whole-brain radiation therapy (WBRT), or combinations of these [12,16].

Primary tumor resection (PTR) is controversial in CRC patients with unresectable metastases [17]. PTR enhances the quality of life and minimizes the negative effects of systemic chemotherapy and the risk of complications from the initial tumor, including bleeding, blockage, and perforation [18]. On the other hand, PTR delays the beginning of systemic chemotherapy, with additional delays if complications emerge [19]. Many physicians choose younger and healthier patients for surgical resection of primary tumors based on their clinical experience. Given the low incidence of BMs from CRC, no relevant reports suggest whether these patients can benefit from PTR.

The goal of this study was to evaluate the survival of individuals with synchronous CRC BMs who had their primary tumor surgically removed with that of patients who did not undergo surgery. To the best of our knowledge, this was the first study of the effect of PTR on their survival from CRC BMs.

## 2. Material and Methods

### 2.1. Study Population

Synchronous BMs were defined as brain metastases present at the time of CRC diagnosis. Data on synchronous BMs were obtained from patients registered between 2010 and 2016 in the National Cancer Institute’s SEER database, which was accessible at https://seer.cancer.gov/ (accessed on 15 November 2021).The following clinicopathological variables were included: age (<60, ≥60 years); race (Black, White, other); gender (female, male); tumor grade (well/moderately differentiated, poorly differentiated/undifferentiated, unknown); histological type (adenocarcinoma, other); surgery at the primary site (PTR, non-PTR); carcinoembryonic antigen (CEA) level (negative, positive, unknown); T stage (T0/T1, T2, T3, T4, unknown); N stage (N0, N1, N2, unknown); M stage (M1a, M1b, unknow) and survival time. The primary tumor location was classified according to CRC subtype, which included right colon cancer (RCC: cecum, ascending colon, hepatic flexure, and transverse colon), left colon cancer (LCC: splenic flexure, descending colon, and sigmoid colon), and rectal cancer (RC: rectosigmoid junction and rectum). The presence of bone, lung, and liver metastases at diagnosis was recorded in the SEER database as the number of extracranial metastases.

**Figure 1:**
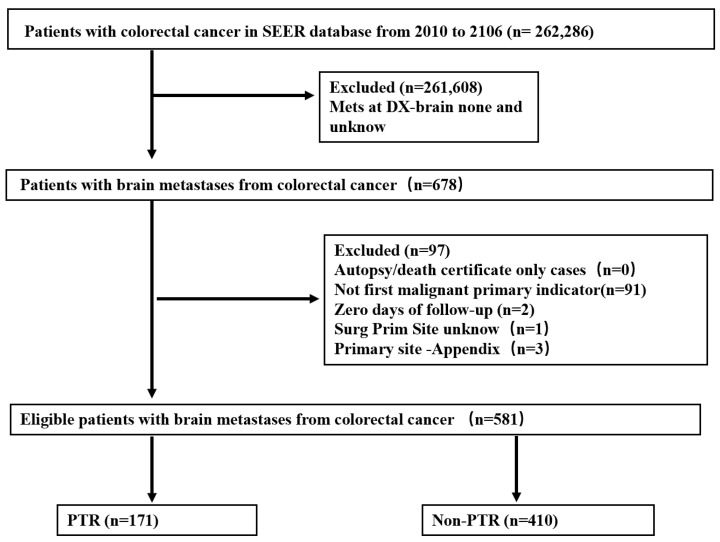
Flow diagram of eligible CRC patients with BMs who had their primary tumor surgically resected or did not have surgery.

Patients were followed from the time of CRC diagnosis until the last follow-up, death, or trial conclusion, whichever came first. Patients with unidentified BMs were excluded from the study, as were those in whom BMs were detected 6 months after CRC diagnosis or during autopsy. The candidate selection technique is depicted in detail in Figure 1.

### 2.2. Statistical Analysis

The chi-square test and Fisher’s exact test were used to compare categorical variables that were given as a number with a percentage. The Kaplan-Meier method and log-rank test were used to generate survival curves. The prognostic variables of patients with brain metastases were determined using Cox proportional hazards models. Univariately significant variables were incorporated into a multivariate analysis to identify independent prognostic factors. A *p*-value < 0.05 was considered statistically significant. SPSS version 25 and Graph Pad Prism 8 were used to execute all statistical procedures.

## 3. Results

### 3.1. Patient Characteristics

Between 2010 and 2016, among 262,286 patients diagnosed with CRC, synchronous BMs were detected in 678, corresponding to an incidence of 0.26%. After rigorous screening, 581 CRC patients with BMs were included (Figure 1). The PTR group contained 171 patients (29.4%), whereas the non-PTR group had 410 patients (70.6%). In terms of primary tumor location, tumor grade, extrahepatic disease, T-stage, N-stage, and CEA, there was a substantial difference between the two groups. Some patients in the non-PTR group had uncertain T-stage, N-stage, or tumor differentiation, which accounted for a significant fraction of the total. Additionally, we discovered that the PTR group had a higher proportion of patients with RCC, M1a, and CEA negative. Of 581 patients, 206 (35.5%) had RCC, 115 (19.8%) had LCC, 170 (29.3%) had RC, and in 90 (15.5%) the CRC type was unknown. Among the total cohort of 581 patients with synchronous BMs, 410 (70.6%) had extracranial metastasis. The patient characteristics are presented in detail in Table 1.

### 3.2. Survival and Prognostic Factors

**Figure 2:**
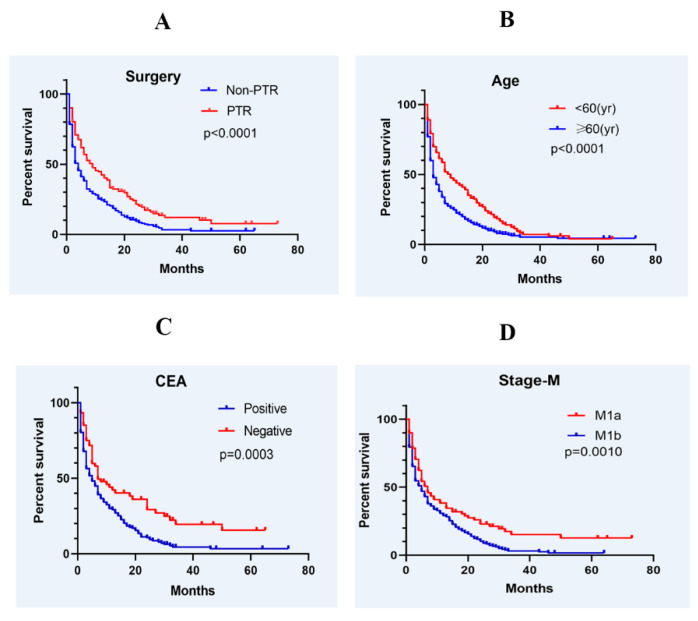
Kaplan-Meier survival curve analysis for overall survival in CRC patients with synchronous BMs. Surgery (**A**), Age (**B**), CEA level (**C**), and extracranial metastases (**D**) were included in the analysis.

Patients with CRC and synchronous BMs had a median OS of 3 months, with a 1-year survival rate of 27.2% and a 2-year survival rate of 12.8%. Patients who underwent primary tumor resection had a 1-year survival rate of 40.2% compared to 21.7% in those who did not (*p* < 0.0001; Figure 2A). Median survival was 9 months in the PTR group, compared with 4 months in the non-PTR group. A comparison of OS by age (<60 and ≥60 years) showed that median survival times were 9 months and 3 months, respectively. The difference in OS between the two groups was statistically significant (*p* < 0.0001; Figure 2B). The 1-year survival rate in the CEA-positive group was 26.7%, much lower than that of the CEA-negative group (42%); the difference was statistically significant (*p* = 0.0003; Figure 2C). Patients without extracranial metastasis also had better outcomes than those with extracranial metastasis (*p* = 0.001; Figure 2D)

In univariate analysis, age, CEA level, primary surgical site, and extracranial metastasis impacted OS in CRC patients with synchronous BMs (Table 2). However, race, sex, primary tumor location, tumor grade, histological types, T stage, and N stage were insignificant.

A multivariate analysis incorporating the significant variables showed that age, CEA level, and PTR were independent prognostic factors (Table 3). Patients ≥ 60 years (hazard ratio [HR] 1.718, 95% confidence interval [CI] 1.423–2.075, *p* < 0.0001) had a shorter OS than patients < 60 years of age. OS was better in CEA-negative than in CEA-positive patients (HR 0.652, 95% CI 0.472–0.899, *p* = 0.009). Similarly, patients in whom the primary tumor was removed had a considerably improved prognosis (HR 0.654, 95% CI 0.531–0.805, *p* < 0.0001).

**Figure 3:**
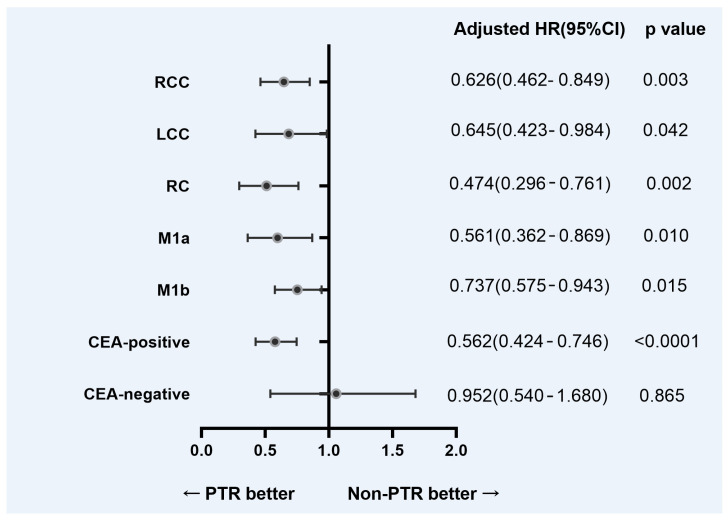
Adjusted hazard ratios for overall survival from the subgroup analysis. Abbreviation: PTR = primary tumor resection; RCC = right colon cancer; LCC = left colon cancer; RC = rectal cancer.

To balance the selection bias between the PTR and non-PTR groups, we further analyzed the corresponding subgroups. Figure 3 shows the adjusted HRs for OS based on the location of the primary tumor, extracranial metastases, and serum CEA level from the subgroup analysis. Except for CEA negative patients, other subgroups of patients with primary tumor resection achieved a survival advantage.

## 4. Discussion

Brain metastases (BMs) in CRC are uncommon, with an incidence of only 0.3–9% [3,8,20]. The somewhat lower incidence in our cohort (0.26%) can be explained by the fact that our study was limited to synchronous brain metastases, which account for 3.4–43% of brain metastases in CRC [6,20]. The prognosis of CRC patients with brain metastases is very poor, as the median survival is only 3 months (range: 2–9.6 months) [3,8,13,21,22,23,24,25]. Understanding the prognostic variables for BMs is critical for determining survival and treatment options. However, there have been very few studies, and a consensus on treatment for BMs in CRC is lacking. From 2010 to 2016, the SEER database included 581 CRC patients with synchronous BMs. According to a multivariate Cox regression analysis performed in this study, age, CEA level, and Primary tumor resection were independent risk factors for synchronous BMs in CRC.

A growing amount of research suggests that patients who have their primary tumor removed have a better chance of surviving. In epithelial ovarian and renal cancers, improved survival results linked with surgical debulking have a well established data foundation [26,27]. However, in advanced CRC, the survival benefit of the PTR is debatable, and current NCCN guidelines support PTR only when symptoms are present [28]. According to some clinicians, the improvement in OS is unclear, such that the morbidity and mortality resulting from tumor resection should be avoided as it will delay the start of chemotherapy, which may reduce survival [19,29]. However, some studies have shown that primary tumor resection considerably improves OS [30,31], as demonstrated in our cohort of CRC patients with synchronous brain metastases. In the primary tumor resection group of this study, the 1-year survival rate was 38.8% compared to 20.9% in patients with unresected primary tumors (*p* < 0.0001). To balance the selection bias between the PTR and non-PTR groups, we further analyzed the corresponding subgroups. Our results showed that PTR had a survival benefit regardless of the site of the primary tumor and with or without extracranial metastases. The higher survival rate following PTR can be related to the decrease of cancer stem cells resistant to chemotherapy and the reduction of primary tumor load [32]. PTR significantly lowered the risk of CRC-related complications such as acute bleeding, perforation, and obstruction, which might result in increased surgical mortality and morbidity [33]. Our forest plot of subgroup analysis revealed that PTR improved survival in CEA-positive CRC BMs patients, but it did not affect CEA-negative patients. This suggests that we should be more cautious about whether to perform surgery for the primary tumor in patients with CRC BMs complicated with CEA negative.

Our results also identified age as a significant prognostic factor for brain metastases in CRC. Patients in our study were divided into two age groups: <60 and ≥60 years. Patients < 60 years of age had a better prognosis. Yang et al. [34] found a considerably poorer prognosis in patients > 70 years than in those 40 years of age. Quan et al. [13] also defined three age groups among CRC patients: <60, 60–74, and ≥75 years. Similar to our findings, patients ≥ 75 years of age had the poorest prognosis, and those < 60 years had the best prognosis. In another study, older age was also identified as an unfavorable prognostic factor for BMs in CRC [35]. Because the prognosis of patients with CRC and synchronous BMs worsens with age, younger individuals should be treated more aggressively than older patients.

The tumor marker CEA is frequently used to monitor CRC patients during treatment. Among patients with metastatic disease, CEA levels are increased (>5 ng/mL) by ~70% [36,37]. Several studies have reported higher CEA levels at the time of brain metastasis diagnosis in CRC patients [38,39,40,41], but only three reported a putative predictive effect [6,13,42]. Consistent with those reports, our study identified CEA as an independent prognostic predictor, with a 1-year survival rate of 26.8% for CEA-positive patients and 40.3% for CEA-negative patients. Thus, CEA positivity is a poor prognostic indicator in CRC patients with brain metastases.

According to our findings, the location of the primary tumor had no prognostic impact in CRC patients with brain metastases. In a univariate analysis, Huerta et al. [43] found that patients with RCC had a poorer survival rate than those with LCC (4.6 vs. 10.7 months; HR 3.5, *p* = 0.025), but in a multivariate Cox regression analysis, the difference was not significant. Another study found that CRC BMs patients with a left-sided primary tumor had a 1.5-fold better prognosis than those with a right-sided primary [14]. Right-sided CRC has a poorer prognosis than left-sided CRC, as evidenced by a greater prevalence of mucinous, undifferentiated, and signet-ring cell tumors, as well as a later stage of cancer at diagnosis [44,45,46]. The lack of a significant link in our study is most likely because it only focused on synchronous BMs. But a recent meta-analysis revealed that the primary CRC site had little impact on the OS [12]. The effect of primary tumor location on the prognosis of patients with BMs of CRC needs further study. In the univariate Cox regression performed in our study, extracranial metastases were significantly related to a poor prognosis, but this result was not confirmed in the multivariate Cox regression. Patients with extracranial metastases and numerous BMs have had poor survival results in prior studies [3,47,48,49]. The survival rate was reduced by up to two-thirds as brain lesions increased [10,11]. CRC patients with synchronous BMs may have a different prognosis risk than those with metachronous BMs. Patients with CRC BMs with a KPS score > 70 had a 4–7 months survival benefit over those with a KPS score < 70 [50,51,52]. Radiation, surgery, chemotherapy, or a combination of the three were the most prevalent treatments for BMs. Patients who had brain metastasectomy had a longer overall survival time than those who had radiotherapy [12]. Metastasectomy or multiagent chemotherapy has been linked to a higher overall survival rate [53]. Survival was reduced by approximately 80% in BMs that were not amenable to targeted treatment [11]. The presence of PD-L1 in the primary tumor was linked to a reduced chance of survival, whereas RAS and BRAF status were not [49].

Our study had several limitations. First, only patients with synchronous BMs with CRC were included because the SEER database only records the status of BMs at the time of initial diagnosis. Second, the SEER database does not contain information on KPS, the number of BMs, comprehensive treatment of BMs, or molecular markers; hence, these parameters were not included in our analysis.

In conclusion, primary tumor resection (PTR) offers survival benefits to patients with synchronous BMs from CRC. Age, CEA level, and PTR were identified as independent risk factors affecting the survival of CRC patients with synchronous BMs.

## Figures and Tables

**Table 1:** Baseline Characteristics of Patients Between the PTR group and the non-PTR group.

Variable	Total (*n* = 581)		Non-PTR (*n* = 410)		PTR (*n* = 171)		*p* Value
**Age (yr)** <60 ≥60	225 356	(37.8) (61.3)	161 249	(39.3) (60.7)	64 107	(37.4) (62.6)	0.678
**Race** Black White Other	67 470 44	(11.5) (80.9) (7.6)	49 328 33	(12.0) (80.0) (8.0)	18 142 11	(10.5) (83.0) (6.4)	0.682
**Gender** Female Male	275 306	(47.3) (52.7)	196 214	(47.8) (52.2)	79 92	(46.2) (53.8)	0.724
**Primary tumor site** RCC LCC RC Unknown	206 115 170 90	(35.5) (19.8) (29.3) (15.5)	115 70 142 83	(28.0) (17.1) (34.6) (20.2)	91 45 28 7	(53.2) (26.3) (16.4) (4.1)	<0.0001
**Histology** Adenocarcinoma Other	505 76	(86.9) (13.1)	357 53	(87.1) (12.9)	148 23	(86.5) (13.5)	0.865
**Tumor grade** Well/moderately Poorly/undifferentiated Unknown	222 137 222	(38.2) (23.6) (38.2)	129 69 212	(31.5) (16.8) (51.7)	93 68 10	(54.4) (39.8) (5.8)	<0.0001
**T stage** T0/T1 T2 T3 T4 Tx	64 20 132 100 265	(11.0) (3.4) (22.7) (17.2) (45.6)	61 12 44 35 258	(14.9) (2.9) (10.7) (8.5) (62.9)	3 8 88 65 7	(1.8) (4.7) (51.5) (38.0) (4.1)	<0.0001
**N stage** N0 N1 N2 Nx	192 162 90 137	(33.0) (27.9) (15.5) (23.6)	154 105 17 134	(37.6) (25.6) (4,1) (32.7)	38 57 73 3	(22.2) (33.3) (42.7) (1.8)	<0.0001
**M stage** M1a M1b M1x	106 410 65	(18.2) (70.6) (11.2)	55 309 46	(13.4) (75.4) (11.2)	51 101 19	(29.8) (59.1) (11.1)	<0.0001
**CEA** Positive Negative Unknown	309 64 208	(53.2) (11.0) (35.8)	222 32 156	(54.1) (7.8) (38.0)	87 32 52	(50.9) (18.7) (30.4)	<0.0001

Abbreviations: PTR, primary tumor resection; RCC, right colon cancer; LCC, left colon cancer; RC, rectal cancer.

**Table 2:** Univariate Cox regression for overall survival among patients with brain metastases.

Clinicopathologic Variable	HR	95%CI	*p* Value
**Age (yr)** <60 ≥60	Reference 1.708	1.418–2.057	<0.0001
**Race** Black White Other	Reference 0.903 0.972	0.693–1.176 0.648–1.458	0.447 0.893
**Gender** Female Male	Reference 1.087	0.911–1.297	0.356
**Primary tumor site** RCC LCC RC Unknown	Reference 0.840 0.838 1.236	0.655–1.078 0.671–1.046 0.949–1.611	0.171 0.118 0.116
**Histology** Adenocarcinoma Other	Reference 1.090	0.840–1.414	0.518
**Tumor grade** Well/moderately Poorly/undifferentiated Unknown	Reference 1.118 1.432	0.884–1.413 1.170–1.751	0.352 <0.0001
**T stage** T0/T1 T2 T3 T4 Tx	Reference 1.219 0.886 1.171 1.336	0.721–2.063 0.634–1.237 0.831–1.651 0.988–1.806	0.460 0.476 0.366 0.060
**N stage** N0 N1 N2 Nx	Reference 1.006 0.863 1.242	0.801–1.264 0.655–1.137 0.980–1.573	0.958 0.295 0.073
**M stage** M1a M1b M1x	Reference 1.393 1.349	1.094–1.772 0.959–1.898	0.007 0.086
**CEA** Positive Negative Unknown	Reference 0.556 1.167	0.406–0.759 0.965–1.410	<0.0001 0.110
**Surgery** Non-PTR PTR	Reference 0.629	0.515–0.768	<0.0001

Abbreviations: PTR, primary tumor resection; RCC, right colon cancer; LCC, left colon cancer; RC, rectal cancer; CI, confidence interval; HR, hazard ratio.

**Table 3:** Multivariable Cox regression for overall survival among patients with brain metastases.

Clinicopathologic Variable	HR	95%CI	*p* Value
**Age (yr)** <60 ≥60	Reference 1.718	1.423–2.075	<0.0001
**M stage** M1a M1b M1x	Reference 1.167 1.020	0.905–1.506 0.716–1.454	0.234 0.912
**CEA** Positive Negative Unknown	Reference 0.652 1.140	0.472–0.899 0.935–1.389	0.009 0.196
**Surgery** Non-PTR PTR	Reference 0.654	0.531–0.805	<0.0001

Abbreviations: PTR, primary tumor resection; CI, confidence interval; HR, hazard ratio.

## Data Availability

The data that support the findings of this study are openly available in the Surveillance, Epidemiology, and End Results (SEER) database of the National Cancer Institute at https://seer.cancer.gov/ (accessed on 15 November 2021).

## Abbreviations

CRC	colorectal cancer
BMs	brain metastases
SEER	Surveillance, Epidemiology, and End Results
CEA	carcinoembryonic antigen
RCC	right colon cancer
LCC	left colon cancer
RC	rectal cancer
CI	confidence interval
